# Shock wave lithotripsy, for the treatment of kidney stones, results in changes to routine blood tests and novel biomarkers: a prospective clinical pilot-study

**DOI:** 10.1186/s40001-020-00417-2

**Published:** 2020-06-01

**Authors:** Stephen F. Hughes, Nathan Jones, Samantha J. Thomas-Wright, Joseph Banwell, Alyson J. Moyes, Iqbal Shergill

**Affiliations:** 1 North Wales Clinical Research Centre, Betsi Cadwaladr University Health Board (BCUHB) Wrexham Maelor Hospital, Wrexham, Wales, UK; 2 North Wales & North West Urological Research Centre, Betsi Cadwaladr University Health Board (BCUHB) Wrexham Maelor Hospital, Wrexham, Wales, UK; 3 Department of Biological Sciences, University of Chester, Chester, UK; 4 Department of Haematology, Countess of Chester Hospital, Chester, UK; 5 Department of Blood Sciences, BCUHB Wrexham Maelor Hospital, Wrexham, Wales, UK; 6 School of Medical Sciences, Bangor University, Bangor, Wales, UK; 7 The Alan de Bolla Department of Urology, BCUHB Wrexham Maelor Hospital, Wrexham, Wales, UK

**Keywords:** Shock wave lithotripsy (SWL), Biomarkers, Kidney stones, Inflammation, Routine blood tests, Acute kidney injury (AKI)

## Abstract

**Background:**

The number of patients undergoing shock wave lithotripsy (SWL) for kidney stones is increasing annually, and as such the development of post-operative complications, such as haematuria and acute kidney injury (AKI) following SWL, is likely to increase. The aim of the study was to evaluate changes in routine blood and novel biomarkers following SWL, for the treatment of kidney stones.

**Methods:**

Twelve patients undergoing SWL for solitary unilateral kidney stones were recruited. From patients (8 males and 4 females) aged between 31 and 72 years (median 43 years), venous blood samples were collected pre-operatively (baseline), at 30, 120 and 240 min post-operatively. Routine blood tests were performed using a Sysmex XE-5000, and Beckman Coulter AU5800 and AU680 analysers. NGAL, IL-18, IL-6, TNF-α, IL-10 and IL-8 concentrations were determined using commercially available ELISA kits.

**Results:**

Significant (*p* ≤ 0.05) changes were observed in several blood parameters following SWL. NGAL concentration significantly increased, with values peaking at 30 min post-treatment (*p* = 0.033). Although IL-18 concentration increased, these changes were not significant (*p* = 0.116). IL-6 revealed a statistically significant rise from pre-operative up to 4 h post-operatively (*p* < 0.001), whilst TNF-α significantly increased, peaking at 30 min post-SWL (*p* = 0.05). There were no significant changes to IL-10 and IL-8 concentrations post-SWL (*p* > 0.05).

**Conclusions:**

Changes to routine blood tests and specific biomarkers, in the future, may be more useful for clinicians. In turn, identification of a panel of biomarkers could provide valuable data on “normal” physiological response after lithotripsy. Ultimately, studies could be expanded to identify or predict those patients at increased risk of developing post-operative complications, such as acute kidney injury or. These studies, however, need validating involving larger cohorts.

## Background

Kidney stones are becoming an increasing clinical and economic burden on global health services [1]. Shock wave lithotripsy (SWL) allows a non-invasive treatment of kidney stones smaller than 2 cm [2]. Recent evidence suggests that SWL treatments are being used more frequently, as there has been a 2/3 increase in the prevalence of kidney stones in the UK over a 10-year period [3].

Despite the use of radiological imaging to accurately direct shock waves to the location of the kidney stone, there is still a risk of localised complications associated with SWL [4]. The most common SWL-associated injury is primary vascular haemorrhage [5]. Originally, it was thought that the prevalence of renal haematoma in patients post-operatively was < 1%, however, it was later discovered that 29% of patients developed haematomas post-SWL [5, 6]. Renal trauma also results in an inflammatory response that initiates tissue remodelling and can result in the production of scar tissue [7]. Research has shown that dose-dependant renal fibrosis occurred in canine subjects undergoing SWL [8]. Fibrosis of renal tissue can result in a partial or complete loss of function to the affected area. One study reports that ~ 10% of SWL patient can experience a urinary tract infection after treatment [9]. The overall complication rate, according to CROES URS study, was up to 25%, which included haemorrhage, pyrexia, urine infection (UTI), acute kidney injury (AKI) and sepsis as the most common post-operative complications [10]. However, other potentially devastating injuries following SWL have been reported, including acute pancreatitis, splenic rupture, bowel injury with perforation, myocardial infarction, and rupture of abdominal aortic aneurysms [11–14]. In addition, it is expected that there will be a greater incidence, more recurrent episodes and overall higher number of patients having SWL. Subsequently, the complication rate is likely to increase, especially with an aging society. As such, it may be advantageous to identify patients who are at increased risk of developing complications following SWL.

Current practice provides little, if any, knowledge regarding identifying, or predicting, those patients at increased chance of complications. Novel biological parameters have the potential to identify complications such as bleeding, acute kidney injury and infection, which may arise following SWL. Importantly, to date, there is limited research evaluating the pathophysiological effects of SWL on clinical outcome measures. Crucially, at present, there are minimal studies that have reported the impact of SWL on novel biomarkers such as NGAL, IL-18, IL-6, TNF-α, IL-10 and IL-8.

Previously, our group have published outcomes of haemostatic function following SWL and other operative surgeries, and this may provide a good foundation to undertake larger studies that may ultimately determine which patients are at increased risk of haemorrhage following surgeries [15–18]. Moyes et al. [19] documented that changes to several biochemical and haematological parameters occur following flexible ureteroscopy (FURS), for the removal of kidney stones. Specifically, FURS is an invasive procedure employed for the treatment of stone disease. During this study, 4 patients from 40 developed post-operative complications, which resulted in significant changes to several of the routine biochemical and haematological blood tests. The information provided in this paper, in turn, highlights the need and the importance to undertake further research in this area, and to fully understand the ‘normal’ response to these treatments.

Neutrophil gelatinase-associated lipocalin (NGAL) is a protein that is bound to matrix metalloproteinase-9 (MMP-9) in neutrophils [20]. NGAL is generally found in low concentrations in human tissue, but is significantly increased in cases of trauma to kidney, colon, liver and lung tissue [21]. Originally identified as a component of neutrophil granules, it has since been found to be produced by tissues undergoing inflammation [22]. It has been shown in patients with chronic kidney disease (CKD) that NGAL concentrations correlate with the severity of renal impairment; however, it has been found that NGAL concentrations are much higher in patients with AKI compared with CKD [20, 21]. The upregulation of NGAL during AKI, may therefore provide a specific novel biomarker following SWL, which may help predict or identify an abnormal response to treatment, such as AKI.

Cytokine-mediated inflammation has been implicated in the pathogenesis of AKI and chronic kidney CKD, where endothelial and tissue injuries are associated with the release of specific mediators that may initiate the inflammatory cascade [23, 24]. Interleukin 18 (IL-18) is a pro-inflammatory cytokine, which is upregulated during an inflammatory response. The majority of IL-18 expressed is from activated macrophages; however, IL-18 has also been shown to be expressed in renal tubular epithelial cells [25]. Urine IL-18 concentrations have been found to be significantly raised in patients with AKI compared to those with urinary tract infection, chronic renal insufficiency and nephrotic syndrome [26]. This demonstrates that IL-18 is upregulated in cases of AKI rather than CKD, making it a possible biomarker for the identification of acute injuries following SWL.

Interleukin-6 (IL 6) is a typical example of a multifunctional cytokine involved in the regulation of the immune response, haematopoiesis, and inflammation. Raised serum levels of IL-6 have been associated with sepsis in AKI patients [23, 24]. One of the principal acute-phase cytokines produced by monocytes and macrophages in response to infection is tumour necrosis factor-alpha (TNF-α). TNF-α has a complex and extensive repertoire of functions within the inflammatory cascade system [27]. Increased levels of TNF-α are thought to potentially prime and therefore elicit a more rapid and prominent response from neutrophils during the inflammatory process and may therefore be appreciated to be a noble biomarker for evaluating sustained inflammation or predicting complications [28].

Predominantly, interleukin-8 (IL-8) is a cytokine, comes from leukocytes and many types of cellular tissues. Neutrophils are a major specific target for IL-8 action. IL-8 is routinely being used as a marker for various clinical conditions and is associated with chronic diseases, infections and inflammation [29, 30]. IL-8 has also been reported to be detected in the urine of patients with several inflammatory renal disorders including pyelonephritis, haemolytic uraemic syndrome, graft rejection and various forms of glomerulonephritis [31].

IL-10 levels appear to have been overlooked in urology studies, with information regarding this biomarker and its relation to kidney damage being vague. IL-10 has been reported to play an integral role with respect to infection, as well as inflammation, due to its anti-inflammatory effect [30]. However, as previously mentioned this cytokine is also affected by many other factors. Serum, peritoneal fluid and saliva levels of IL-10 are known to be elevated in conditions such as infections, melanoma, tumours, and autoimmune diseases [32, 33].

As highlighted above, studies involving cytokines in various clinical settings have been well documented. However, with respect to urology, there are limited studies evaluating the role of cytokines following kidney stone treatment and are therefore worthy of investigation.

We aimed to evaluate changes in routine haematological and biochemical blood tests, including novel biological parameters, namely NGAL, IL-18, IL-6, TNF-α, IL-10 and IL-8. This should allow us to understand better, the postoperative biological pathway following SWL, in kidney stone management. This pilot-study has the potential to add a significant body of work to the literature, as well as providing the biological basis for future multi-centre studies.

## Methods

### Subject volunteers and shock wave lithotripsy (SWL)

Following ethical approval (Integrated Research Application System REC 4:12/WA0117), and written informed consent, we recruited 12 patients who underwent SWL for kidney stone treatment. Of these, 8 were male, and the remaining 4 were female. The median age was 43 years (range 31 to 72). Using standard hospital protocol, SWL therapy was delivered with Wolf P3000 lithotripter, incorporating triple focus technology, and ultrasound/fluoroscopic imaging was used for localisation.

### Blood samples

Baseline (control) samples of venous blood were taken before SWL, using a cannula inserted into the ante-cubital fossa for each patient. Subsequent sampling was undertaken at 30, 120 and 240 min post-operatively. Trained healthcare staff were present throughout the study period ensuring that blood samples were collected at the specific time-points. Vacutainers containing di-potassium ethylene diamine tetra-acetic acid (EDTA) were used for sample collection. Plasma was obtained by centrifugation at 1000*g* for 15 min. Plasma was removed from the vacutainer and stored in aliquot tubes at − 80 °C until required for analysis.

An 87.5% compliance was obtained regarding blood sample collection. Participants 2, 7 and 10 (12.5% of the total participants) were unable to provide blood samples at 120 and 240 min post-operatively due to difficulty (i.e. poor veins) with the venesection.

### Measurement of haematological and biochemical parameters

Full blood count (FBC) was undertaken via a Sysmex XE-5000 automated cell counter, and biochemistry tests undertaken using the Beckman Coulter AU5800 and AU680 analysers.

### Measurement of NGAL, IL-18, IL-6, TNF-α, L-10 and IL-8 concentrations

Commercially available Human Quantikine^®^ ELISA kits for NGAL, IL-18, IL-6, TNF-α, IL-10 and IL-8 were purchased from R&D Systems^®^ catalogue numbers: DLCN20, 7620, D6050, DTA00C, D1000B and D8000C, respectively. All assays were run as per manufacturer’s instructions, in duplicate, with sample analysis being undertaken when enough patients were recruited on to the study to run a single 96-well assay plate (*n* = 3 plates per biomarker). Intra-assay % coefficient of variability (CV) levels are reported, with a CV of < 10% being acceptable, ensuring assay precision and high performance. Biomarker assay specifications are illustrated in Table 1.

**Table 1:** Biomarker assay specifications

Biomarker ELISA assay	Assay range (limits of detection)	Sensitivity	Specificity
NGAL	0.2–10 ng/ml	0.04 ng/ml	Natural and recombinant human lipocalin-2
IL-18	26.6–1700 pg/ml	7.52 pg/ml	Natural and recombinant human total IL-18
IL-6	3.1–300 pg/ml	0.7 pg/ml	Natural and recombinant human IL-6
TNF-α	15.6–1000 pg/ml	5.5 pg/ml	Natural and recombinant human TNF-alpha
IL-10	7.8–500 pg/ml	3.9 pg/ml	Natural and recombinant human IL-10
IL-8	31.2–2000 pg/ml	7.5 pg/ml	Natural and recombinant human IL-8

### Statistical analysis

Statistical analysis was carried out using SPSS (latest version). Initial testing for normality was carried out, and where data were parametric, repeated measures analysis of variance (ANOVA) between samples test was employed, adopting a 5% level of significance. Post hoc testing was conducted using the Bonferroni test for pairwise comparisons between means. Data that did not comply with normality were analysed using the Friedman test. Where the Friedman test resulted in statistical significance, subsequent tests were performed using the Wilcoxon test. Statistical significance was accepted when *p* ≤ 0.05. All parametric data are presented as mean ± standard deviation (SD), whilst non-parametric results are presented as median ± interquartile range (IQR), reporting the lower (25 percentile) and upper (75 percentile) bound IQRs.

## Results

### Haematological blood results

Haematological changes following SWL are presented in Table 2. The following biomarkers exhibited statistically significant decreases: basophils (*p* = 0.041), haemoglobin (*p* = 0.002), red blood cells (*p* = 0.001), and packed cell volume (*p* = 0.002). Furthermore, significant increases were seen in white blood cell (WBC) levels (*p* = 0.009), neutrophils (*p* = 0.017), monocytes (*p* = 0.003) and mean corpuscular haemoglobin (*p* = 0.047). No changes were reported in lymphocytes, eosinophils, mean cell volume, and mean corpuscular haemoglobin concentration (*p* > 0.05).

**Table 2:** Haematological changes following SWL (*n* = 12)

	Baseline	30 min	120 min	240 min	Reference range	*p* value	Statistical test
White blood cells (× 10^9^/L)	5.7 (± 4.3/10.10)	7.15 (± 5.2/11.4)*	5.8 (± 5.10/14.2)	8.0 (± 3.9 5.5/14.50)*	4.0–11.0	*0.009*	Friedman
Neutrophils (× 10^9^/L)	3.6 (± 2.4/7.4)	4.05 (± 2.9/8.60)	3.8 (± 2.90/11.90)	5.2 (± 3.4/11.2)*	1.7–7.5	*0.017*	Friedman
Lymphocytes (× 10^9^/L)	1.6(± 1.3/3.6)	1.85 (± 1.3/3.1)	1.50 (± 1.0/2.2)	2.0 ± (1.0/3.0)	1.0–4.5	0.063	Friedman
Eosinophil (× 10^9^/L)	0.1 (± 0.05/0.4)	0.1 (± 0.02/0.5)	0.1 (± 0.03/0.2)	0.1 (± 0.03/0.3)	0.0–0.4	0.101	Friedman
Basophils (× 10^9^/L)	0.025 (± 0.01/0.6)	0.025 ± (0.01/0.6)	0.02 (± 0.01/0.03)	0.02 (± 0.01/0.07)	0.0–0.1	*0.041*	Friedman
Monocytes (× 10^9^/L)	0.5 (± 0.3/0.6)	0.5 (± 0.4/0.8)	0.4 (± 0.3/0.8)	0.8 (± 0.5/1.0)*	0.2–0.8	*0.003*	Friedman
Haemoglobin (g/L)	124.5 (± 123/152)	117.5 (± 120/151)*	124 (± 120/141)*	120 ± (117/141)*	M: 130–180 F: 115–165	*0.002*	Friedman
Red blood cells (× 10^9^/L)	4.84 (± 4.3/5.51)	4.74 (± 4.06/5.27)*	4.62 (± 4.12/4.90)*	4.74 (± 4.23/5.17)*	M: 4.5–6.0 F: 3.8–5.5	*0.001*	Friedman
Mean corpuscular haemoglobin (pg)	28.83 (± 25.4/31.7)	29.1 (± 26.6/32.4)*	29.4 (± 28.7/32.4)*	29.4 (± 27.7/32.1)	27.0–32.0	*0.047*	Friedman
Mean cell volume (fl)	87.3 (± 32.0/106.6)	87.9 (± 32.2/106.2)	88.7 (± 79.6/106.4)	88.7 (± 81.0/105.8)	80.0–100.0	0.404	Friedman
Packed cell volume (L/L)	0.42 ± 0.03	0.41 ± 0.04	0.39 ± 0.02	0.41 ± 0.03	M: 0.4–0.52 F: 0.37–0.47	*0.002*	ANOVA
Mean corpuscular haemoglobin concentration (%)	34.02 ± 1.65	33.95 ± 1.23	34.27 ± 1.12	33.68 ± 0.83	32–36	0.179	ANOVA

Statistical significance following post hoc analysis is represented when *p ≤ 0.05. (M, male; F, female)

### Biochemistry blood results

Biochemical changes following SWL are presented in Table 3. The following exhibited statistically significant decreases: total protein (*p* = < 0.001), albumin (*p* = < 0.001), globulin (*p* = 0.006), alkaline phosphatase (*p *= 0.018) and sodium (*p* = 0.01). Furthermore, significant increases were seen in alkaline transaminase (*p* = 0.01). No changes were reported in CRP, total bilirubin, urea, creatinine, and potassium (*p* > 0.05).

**Table 3:** Biochemical changes following SWL (*n* = 12)

	Baseline	30 min	120 min	240 min	Reference range	*p* value	Statistical test
CRP (mg/L)	3.1 (± 1.0/8.0)	1.65 (± 1.0/7.0)	1.8 (± 1.0/7.5)	1.9 (± 1.0/6.40)	0–5.0	0.101	Friedman
Total protein (g/L)	71.33 ± 4.83	67.93 ± 4.23*	64.45 ± 3.45*	67.64 ± 3.03*	60.0–80.0	*< 0.001*	ANOVA
Albumin (g/L)	43.13 ± 3.0	40.90 ± 3.12*	39.33 ± 3.5*	40.77 ± 3.22	35.0–45.0	*< 0.001*	ANOVA
Globulin (g/L)	28 (± 25/37)	28 (± 22/30)	26 (± 23/27)*	26.5 (± 25/30)	23.0–35.0	*0.006*	Friedman
Total bilirubin (µmol/L)	13.5 (± 7/23)	15 (± 7/22)	12 (± 9/43)	11 (± 8/22)	< 21	0.140	Friedman
Alkaline phosphatase—ALP (U/L)	73 (± 36/91)	65 (± 37/93)*	68.5 (± 35/85)*	72.5 (± 34/86)	30.0–130.0	*0.018*	Friedman
Alkaline transaminase—ALT (U/L)	35.5 (± 18/70)	34 (± 18/65)	35 (± 17/62)	37 (± 15/67)*	< 41 (male) < 33 (female)	*0.01*	Friedman
Urea (mmol/L)	4.90 (± 2.7/6.0)	4.80 (± 2.8/6.0)	4.90 (± 3.6/5.20)	4.85 (± 3.30/5.20)	2.5–7.8	0.478	Friedman
Creatinine (µmol/L)	67.1 (± 11.39)	69.7 (± 13.04)	73.0 (± 12.30)	71.1(± 14.09)	M: 58.0–110.0 F: 46.0–92.0	0.365	ANOVA
Sodium (mmol/L)	140 (± 135/140)	139 (± 132/143)*	137 (± 134/142)*	138 (± 133/140)*	135.0–146.0	*0.010*	Friedman
Potassium (mmol/L)	4.27 (± 3.9/4.6)	4.37 (± 3.64/4.60)	4.12(± 3.4/5.0)	4.2 (± 3.8/4.60)	3.5–5.3	0.291	Friedman

Statistical significance following post hoc analysis is represented when *p ≤ 0.05. (M, male; F, female)

### Novel biomarkers blood results

#### NGAL

**Fig. 1:**
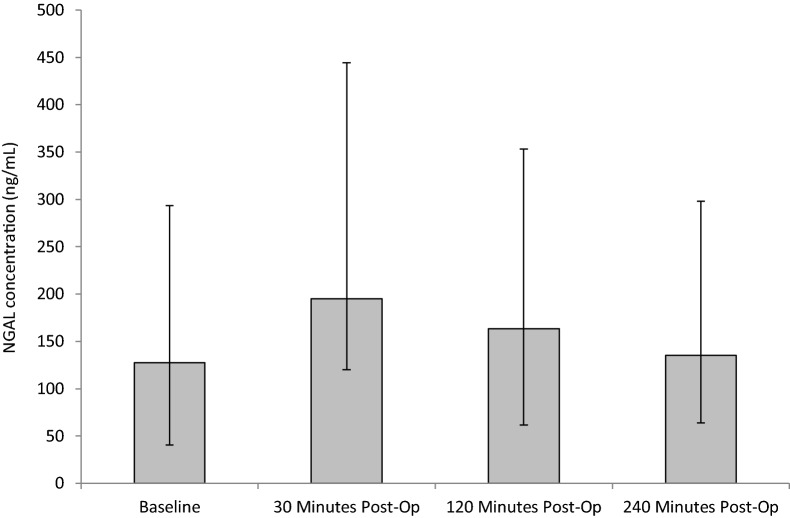
The effect of SWL, for the treatment of kidney stones, on NGAL concentration. Data points expressed as median ± IQR. *p* = 0.033 as determined by Friedman test. The intra-assay CV was 4.8%. Patient samples were diluted 1 in 60 as per manufacturer guides

Plasma NGAL concentration increased from baseline (pre-operative) (127.3 ± 87.4/166.8) and peaked at 30 min (195.1 ± 75.4/249.5) post-SWL (Fig. 1). At 120 min (163.4 ± 102.5/190.1) and 240 min (135 ± 71.1/163.1) NGAL concentration decreased towards basal levels. According to the Friedman test, there were statistically significant rises in NGAL following SWL (*p* = 0.033). Upon further post hoc testing no other changes were observed from baseline to individual time-points (*p* > 0.05).

#### IL-18

**Fig. 2:**
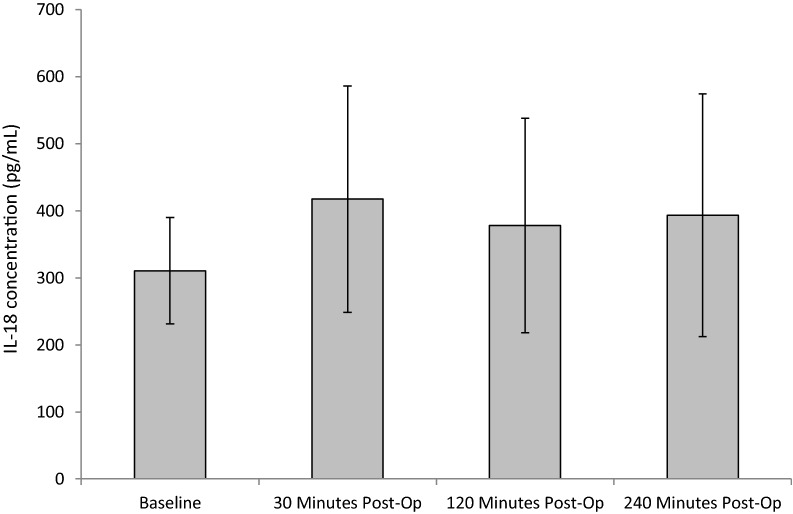
The effect of SWL, for the treatment of kidney stones, on IL-18 concentration. Data points expressed as mean ± standard deviation. *p* = 0.89 as determined by a repeated measures ANOVA. The intra-assay CV was 5.1%

Mean concentrations of plasma IL-18 increased from the baseline (pre-operatively) (310 ± 79.3) and peaked at the initial post-operative sampling of 30 min post-operatively (417.5 ± 168.9) (Fig. 2). Mean concentrations of IL-18 were consistently raised with only slight changes at 120 min (378.2 ± 160) and 240 min (393 ± 181.2). Collectively, there was no significant difference in IL-18 (*p* = 0.116), as determined by ANOVA. Although not statistically significant, there was a trend of increasing IL-18 concentration up to 4 h post-SWL.

#### IL-6

**Fig. 3:**
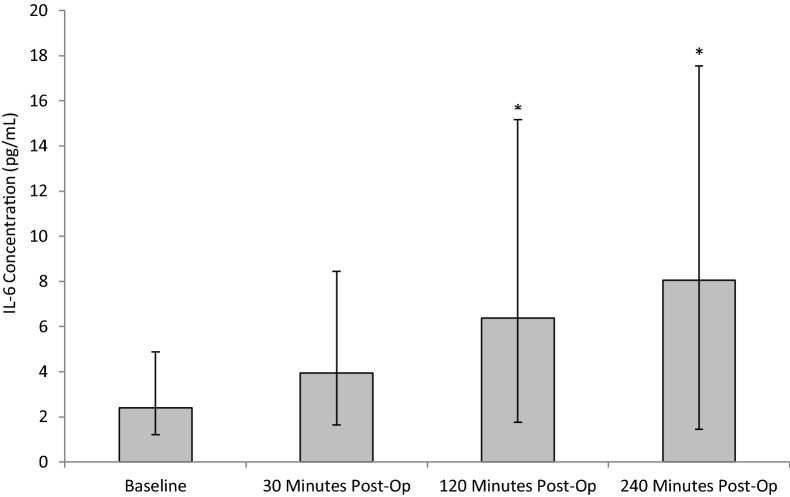
The effect of SWL, for the treatment of kidney stones, on IL-6 concentration. Data points expressed as median ± IQR. *p* < 0.001 as determined by Friedman test. The intra-assay CV was 8.1%

Following SWL, IL-6 levels increased across all time-points. Specifically, IL-6 increased from baseline (pre-operative) (2.40 ± 1.19/2.47), during 30 min (3.94 ± 2.3/4.5), 120 min (6.37 ± 4.6/8.8) and 240 min (8.05 ± 6.6/9.5) post-operatively (Fig. 3). Statistical significance was determined using the Friedman test (*p* < 0.01). Upon further testing using the Wilcoxon test a statistically significant difference was shown between the baseline value and 120 min, 240 min post-operatively (*p* = 0.013, *p* = 0.05, respectively).

#### TNF-α

**Fig. 4:**
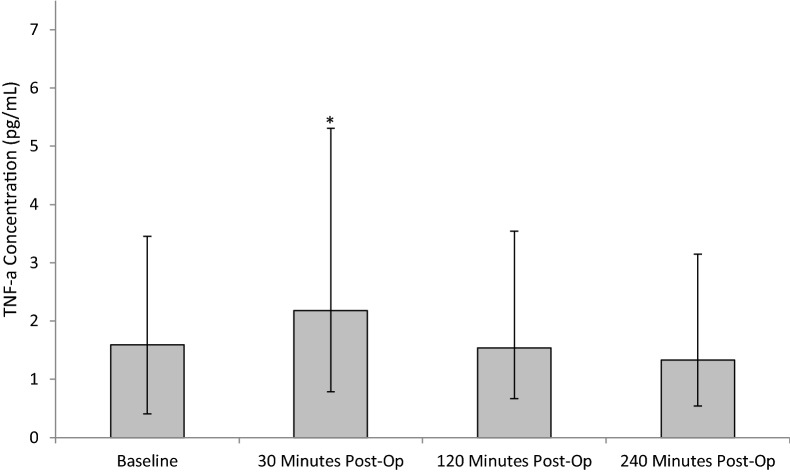
The effect of SWL, for the treatment of kidney stones, on TNF-α concentration. Data points expressed as median ± IQRr. *p* = 0.05 as determined by Friedman test. The intra-assay CV was 6.5%

Following SWL, TNF-α levels increased from baseline (pre-operative) (1.592 ± 0.26/4.75) and peaking at 30 min post-operatively (2.18 ± 0.67/2.18). At 120 min (1.54 ± 0.39/3.69) and 240 min (1.33 ± 0.3/3.56) TNF-α concentrations decreased (Fig. 4). Statistical significance was determined using the Friedman test (*p* = 0.05). Upon further testing using the Wilcoxon test, significant difference was shown between baseline value and 30 min post-operatively (*p* = 0.041).

#### IL-10

**Fig. 5:**
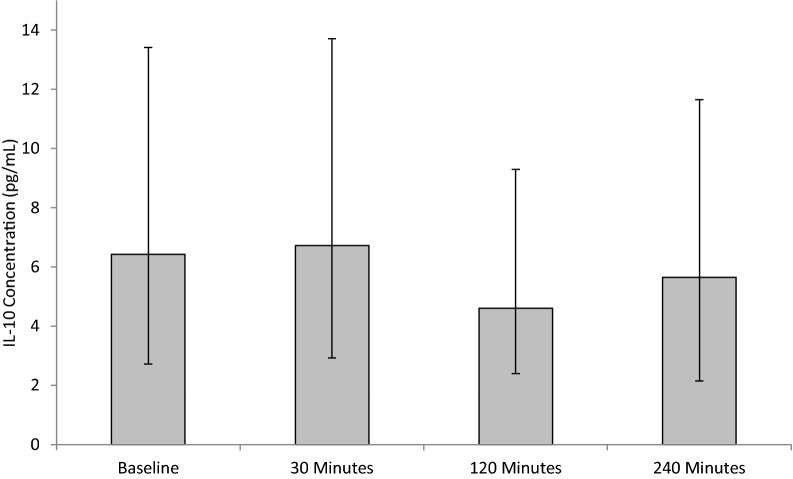
The effect of SWL, for the treatment of kidney stones, on IL-10 concentration. Data points expressed as median ± IQRr. *p* = 0.086 as determined by Friedman test. The intra-assay CV was 8.1%

Following SWL no significant changes were seen in IL-10 concentration, *p*= 0.086 as determined by the Friedman test (Fig. 5). Although no significant changes were observed, IL-10 concentrations slightly increased from baseline (6.43 ± 3.7/7.0), peaking at 30 min (6.72 ± 3.8/7.2) post-operatively. Following 120 (4.6 ± 2.2/4.7) and 240 min (5.65 ± 3.5/6.1) postoperatively levels decreased.

#### IL-8

**Fig. 6:**
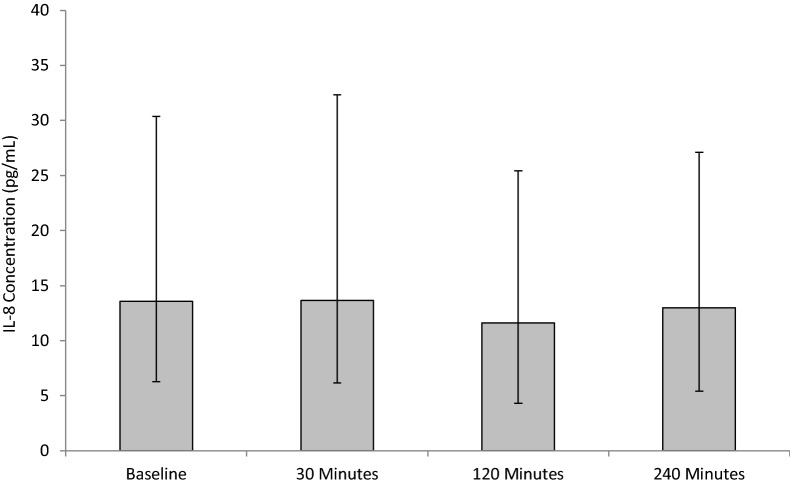
The effect of SWL, for the treatment of kidney stones, on IL-8 concentration. Data points expressed as median ± IQRr. *p* = 0.187 as determined by Friedman test. The intra-assay CV was 5.1%

Following SWL, no significant changes were seen in IL-8 concentrations, *p* = 0.187 as determined by the Friedman test (Fig. 6). Although non-significant, IL-8 concentrations slightly increased from baseline (13.58 ± 7.3/16.8) at 30 min (13.65 ± 7.5/18.7) and decreased thereafter at 120 min (11.63 ± 7.3/13.8), and at 240 min (12.94 ± 7.6/14.1) post-operatively.

## Discussion

The aim of this study was to investigate the effects of SWL, for the treatment of kidney stones, on routine blood tests, and specific biomarkers, namely NGAL, IL-18, IL-6, IL-10 and IL-8. Interestingly, in the current study, no post-operative complications were reported. As SWL is considered a relatively minimally invasive procedure, usually undertaken under local anaesthesia, it can be appreciated that the reported outcomes are directly relatable to the procedure. It can therefore be appreciated that this prospective feasibility study provides crucially important information on “normal” physiological outcomes after SWL.

With regard to routine blood tests, significant changes to several haematological and biochemical parameters were observed following SWL. Interestingly, our findings are like those occurring after other minimally invasive urological surgery for the treatment of kidney stones [19]. In the present study, specifically, total leukocyte (white blood cells), neutrophils, erythrocytes (red blood cells) and haemoglobin concentrations, increased and decreased, respectively; whilst significant decreases in total protein, albumin, globulin, ALP, and sodium (biochemical parameters) were observed.

Changes to haematological markers following upper and lower limb orthopaedic surgical procedures have been reported by others [16, 17]. However, little is known about the role of routine biochemical and haematological blood tests following SWL. Hughes et al. [15] have previously reported changes to fibrinogen and vWF (haemostatic function markers), that may help identify and subsequently predict patients at increased risk of bleeding complications following SWL. Similar observations were reported by Wozniak et al. [18], where it was proposed that oxidative stress may result in haemostatic changes in kidney stone patients, both prior to and especially after SWL, suggesting that SWL modulates haemostasis, and may contribute to coagulopathy episodes that can occur in high-risk patients following SWL.

In addition to the routine blood test results, biomarkers such as NGAL, IL-6 and TNF-α significantly increased following SWL, with the most noticeable changes occurring at 30 min post-operatively for most biomarkers. Devarajan [21] has reported that NGAL provides an excellent biomarker for the early diagnosis of AKI, and for the prediction of clinical outcomes and mortality in several common clinical circumstances. Biologically, it can be appreciated that during SWL, high stresses are placed on the kidney, which may result in AKI, and subsequent impairment of renal function. Serum creatinine has long been considered a biomarker of choice for AKI, although not sensitive and is unreliable. With regard to our present study, creatinine levels increased beyond 2 h post-SWL, whereas NGAL peaked at 30 min and returned towards basal levels between 2 and 4 h. It may therefore be proposed that any sustained increased changes to NGAL may provide a reliable marker for identifying and subsequently monitoring AKI following SWL.

Although there are many variables that can influence NGAL concentration within blood, such as GFR and formation of the biological agent by other cells such as neutrophils, it is important to remember that NGAL is not specific in diagnosing the aetiology of renal impairment, but may apply a crude method of indicating some sort of underlying pathology that needs to be clinically addressed [21, 34, 35]. It could therefore be appreciated that NGAL could act as an indicator, upon which further investigations, including medical imaging, analysis of a panel of biomarkers are undertaken to reveal the full extent of the complications.

With regard to IL-18, Faust et al. [25] and Parikh et al. [26] demonstrated that this pro-inflammatory cytokine is expressed in renal tubular epithelial cells, and urine levels of IL-18 have been reported to be raised in AKI compared to those with other co-morbidities, such as urinary tract infection. Although no significant changes were observed in IL-18 concentration following SWL in the present study, trends of increasing levels were reported, highlighting the need to undertake further studies involving larger cohorts into this novel biological parameter.

Previous studies have reported of raised serum IL-6 levels being associated with sepsis in AKI patients [23, 24]. Our study reports a similar pattern of increasing IL-6 following SWL. Although one can appreciate that further investigations involving a larger cohort would be required, IL-6 may potentially provide an additional biomarker screening tool for predicting the severity of AKI and subsequent urosepsis that may develop in high-risk patients following lithotripsy. A similar pattern of increasing TNF-α concentration was observed following SWL, which affirms along with other studies the integral role that this cytokine plays during an inflammatory response [27, 28]. Although no significant changes were observed in IL-10 and IL-8, trends of increasing concentrations were observed in the present study. These findings agree with others who have demonstrated increased levels in various clinical settings [30, 32, 36].

To date, little is known about the effect of SWL on routine blood tests and novel biomarkers. This pilot-study, we believe, has contributed to the literature, as well as providing the biological basis for future multi-centre studies. We have shown that changes to several biochemical and haematological (routine) blood tests, including specific biomarkers, such as NGAL, occur following SWL. It is hypothesised that if larger multi-centre cohort studies reproduce these findings, then these biomarkers (or probably a panel of biomarkers) may potentially provide clinicians with a better understanding of the “normal” physiological response following SWL, and thus may allow changes in clinical protocols for patient management. For example, any sustained changes to selective biomarkers, may provide clinically useful information, such as identifying or predicting the development of infection or significant bleeding episodes following SWL [13, 15–17].

Clearly, the weaknesses of this study include patient numbers which have been recruited thus far (*n* = 12), subsequent blood sampling opportunities beyond 4 h, and the use of controls. Although the numbers are small, it was designed as a pilot study, and as such, we feel that we have provided a scientific basis to assess these biomarkers and their correlation with clinical outcome, in a larger multi-centre cohort study. Logistically, it was very difficult to retain patients beyond 4 h, as SWL was carried out as a strict day case treatment. Our current ethical approval did not allow SWL to be performed with normal healthy individuals (e.g. control patients, without kidney stones), but in future studies we could compare SWL with kidney stone patients undergoing other minimally invasive therapies. This approach could also allow us to identify and establish the “normal” post-operative physiological response after urological treatment.

## Conclusion

Changes to routine blood tests and specific biomarkers, in the future, may be more useful for clinicians. In turn, identification of a panel of biomarkers could provide valuable data on “normal” physiological response after lithotripsy. Ultimately, studies could be expanded to identify or predict those patients at increased risk of developing post-operative complications, such as acute kidney injury or. These studies, however, need validating involving larger cohorts.

## Data Availability

All data generated or analysed during this study are included in the published article.

## Abbreviations

AKI: Acute kidney injury
EDTA: Ethylene diaminetetraacetic acid
NGAL: Neutrophil gelatinase-associated lipocalin
ESR: Erythrocyte sedimentation rate
IL: Interleukin
TNF-α: Tumour necrosis factor-alpha
SD: Standard deviation
SWL: Shock wave lithotripsy
